# Evaluation of an HIV-Related Mortuary Surveillance System — Nairobi, Kenya, Two Sites, 2015

**DOI:** 10.15585/mmwr.ss6714a1.

**Published:** 2018-12-21

**Authors:** Hammad Ali, Catherine Kiama, Lilly Muthoni, Anthony Waruru, Peter W. Young, Emily Zielinski-Gutierrez, Wanjiru Waruiru, Richelle Hark‎lerode, Andrea A. Kim, Mahesh Swaminathan, Kevin M. De Cock, Joyce Wamicwe

**Affiliations:** 1 Division of Global HIV and Tuberculosis, Center for Global Health, CDC; 2 Field Epidemiology Training Program, Ministry of Health, Nairobi, Kenya; 3 National AIDS and STI Control Programme, Ministry of Health, Nairobi, Kenya; 4 Institute for Global Health Sciences, University of California, San Francisco

## Abstract

**Problem/Condition:**

Use of human immunodeficiency virus (HIV)-mortality surveillance data can help public health officials monitor, evaluate, and improve HIV treatment programs. Many high-income countries have high-coverage civil registration and vital statistics (CRVS) systems linked to case-based HIV surveillance on which to base HIV mortality estimates. However, in the absence of comprehensive CRVS systems in low- and medium-income countries, such as Kenya, mortuary surveillance can be used to understand the occurrence of HIV infection among cadavers. In 2015, a pilot HIV-related mortuary surveillance system was implemented in the two largest mortuaries in Nairobi, Kenya. CDC conducted an evaluation to assess performance attributes and identify strengths and weaknesses of the surveillance system pilot.

**Period Covered:**

Data collection: January 29–March 3, 2015; evaluation: November 2015.

**Description of the System:**

The surveillance system objectives were to determine HIV positivity among cadavers at two mortuary sites in Nairobi, Kenya, and to determine annual cause-specific and HIV-specific mortality rates among the cadavers. Cadavers of persons aged ≥15 years at death admitted to either mortuary during a 33-day period were included. Demographic information and place and time of death were entered into a surveillance register. Cardiac blood was collected using transthoracic aspiration, and blood specimens were tested for HIV in a central laboratory. Causes of death were abstracted from mortuary and hospital records. Of the 807 cadavers brought to the mortuaries, 610 (75.6%) had an HIV test result available. The overall unadjusted HIV-positivity rate was 19.5% (119/610), which differed significantly by sex (14.6% among men versus 29.5% among women).

**Evaluation:**

The evaluation was conducted using CDC guidelines for evaluating public health surveillance systems. The attributes of simplicity, flexibility, data quality (completeness and validity), acceptability, sensitivity, predictive value positive, representativeness, timeliness, and stability were examined. The evaluation steps included review of the surveillance system documents, in-depth interviews with 20 key informants (surveillance system staff, including mortuary and laboratory staff, and stakeholders involved in funding or implementation), and review of the surveillance database.

**Results and Interpretation:**

Implementation of the pilot mortuary surveillance system was complex because of extensive paperwork and the need to collect and process specimens outside of business hours. However, the flexibility of the system accommodated multiple changes during implementation, including changes in specimen collection techniques and data collection tools. Acceptability was initially low among the mortuary staff but increased after concerns regarding workload were resolved. Timeliness of specimen collection could not be measured because time of death was rarely documented. Completeness of data available from the system was generally high except for cause of death (46.5%). Although the two largest mortuaries in Nairobi were included, the surveillance system might not be representative of the Nairobi population. One of the mortuaries was affiliated with the national referral hospital and included cadavers of admitted patients, some deaths might have occurred outside Nairobi, and data were collected for only 1 month.

**Public Health Actions:**

Mortuary surveillance can provide data on HIV positivity among cadavers and HIV-related mortality, which are not available from other sources in most sub-Saharan African countries. Availability of these mortality data will help describe a country’s progress toward achieving epidemic control and achieving Joint United Nations Programme on HIV/AIDS 95-95-95 targets. To understand HIV mortality in high-prevalence regions, the mortuary surveillance system is being replicated in Western Kenya. Although a low-cost system, its sustainability depends on external funding because mortuary surveillance is not yet incorporated into the national AIDS strategic framework in Kenya.

## Introduction

Routine monitoring of mortality is vital for public health planning. Cause-specific death rates are an important indicator of local disease epidemiology (*1*,*2*) and can be used for monitoring trends in population health status (*3*,*4*). Mortality data are an essential component of global health statistics (*5*–*8*). In countries with functioning civil registration and vital statistics (CRVS) systems, CRVS data are considered the gold standard for evaluating changes in mortality (*9*). These countries usually have unique identifiers and electronic reporting that enhance quality and completeness of CRVS. However, many countries in sub-Saharan Africa have weak CRVS systems with poor data quality (*10*,*11*).

Because deaths of persons infected with human immunodeficiency virus (HIV), the virus that causes acquired immunodeficiency syndrome (AIDS), can be a direct outcome of lack of or ineffective treatment, mortality surveillance data are critical to monitoring, evaluating, and improving HIV treatment programs. Advancements in antiretroviral therapy (ART) have resulted in near-normal life expectancy among persons receiving treatment (*12*,*13*). Therefore, monitoring cause of death along with HIV status at time of death is important for programmatic response and HIV surveillance purposes. Even when CRVS systems are in place, the available cause of death data are often incomplete, unreliable, or both, and data are inadequate to track mortality trends (*14*–*16*). Studies from countries including Botswana, Côte d’Ivoire, Mozambique, and the United States have used mortuary-based surveillance (through complete, partial, or minimally invasive autopsies) as an alternative method for obtaining cause of death data (*14*,*17*–*20*). In the absence of adequate data on HIV mortality trends from CRVS systems, mortuary surveillance data can be used to understand the occurrence of HIV infection among cadavers, as highlighted by previous studies (*12*,*18*,*21*).

Kenya has a paper-based birth and death records register, which makes retrieving death records and routinely analyzing these data for mortality trends difficult (*22*). Therefore, Kenya does not report mortality and cause of death data to the World Health Organization (*23*). According to the Kenya Ministry of Health (MOH), HIV is the leading cause of death among adults in the country (*24*). In 2016, the overall national prevalence of HIV infection among persons aged 15–64 years was 5.4% (*25*). 

Kenya has two types of mortuaries: 1) stand-alone mortuaries (i.e., not affiliated with hospitals) and 2) mortuaries affiliated with hospitals. During the admission process, demographic information is entered into a mortuary register for each cadaver. Hospital deaths usually include a death notification completed by an attending physician. A full postmortem autopsy is performed for all cadavers brought in by police, or at the request of family members, according to each mortuary’s standard operating procedures (SOPs), to determine underlying, immediate, and contributing causes of death. An autopsy form is completed, including the type of procedure, tests, and findings, for each cadaver undergoing a full postmortem autopsy. Because of inadequate national-level HIV mortality data, a simple, cost-effective pilot system for HIV-mortality surveillance in mortuaries was implemented in two mortuaries in Nairobi because Nairobi County has the highest number of persons living with HIV in Kenya (*26*).

## HIV-Related Mortuary Surveillance System in Kenya

In 2015, an HIV-related mortuary surveillance system was piloted in Nairobi to determine HIV positivity among cadavers, annual cause-specific mortality rates, and annual HIV-specific mortality rates (*27*,*28*). Sample size calculations were performed to determine the number of specimens required to measure HIV-related deaths with prevalence as low as 5% of all deaths and the approximate duration of data collection. Kenyatta National Hospital (KNH) mortuary and Nairobi City mortuary, the two largest mortuaries ranked by number of cadavers admitted monthly, were included. Most cadavers at KNH mortuary were from KNH, the national referral hospital. Data from cadavers of persons aged ≥15 years at death admitted to either mortuary during a 33-day period (January 29–March 3, 2015) were included in the surveillance system.

For each cadaver, demographic information (age, sex, county of residence, circumstances of death, and place and time of death) was entered into a surveillance register. Cardiac blood was collected from unpreserved cadavers using transthoracic aspiration, and blood specimens were sent to a central laboratory for processing. Cadavers of persons for whom blood could not be collected (i.e., a preserved cadaver or a cadaver whose condition was too deteriorated to allow blood collection) were excluded. Blood was tested for HIV at a central laboratory using the national diagnostic algorithm (*29*): screen specimen for HIV antibody with the Colloidal Gold assay, confirm reactive results with the First Response HIV 1-2-0 card test, and test discrepant results with the Uni-Gold HIV assay. In case of a discrepancy, the Uni-Gold HIV assay result was final. Viral load (HIV RNA copies/mL) testing was done on all samples that tested positive for HIV. At KNH mortuary, for all deaths that occurred at the hospital, records were obtained from the central medical records office. The following information was extracted from the medical records: cause of death (coded according to the *International Classification of Diseases, Tenth Revision* [*ICD-10*] by the hospital’s *ICD-10* unit), diagnosis, symptoms of AIDS, and history of HIV testing and ART use. For either mortuary, if an autopsy was conducted, cause of death was extracted from the autopsy results.

### Summary of Findings from the Surveillance System

Findings from the surveillance system have been published elsewhere (*27*,*28*). In summary, 807 cadavers of persons aged ≥15 years (age range: 15–105 years; median age: 40 years) were brought to the two mortuaries during the 33-day period. Specimens were not available for 167 cadavers for reasons including logistics and staffing (n = 91), extensive burns or putrefaction (n = 26), other/legal/discharge or transfer of cadaver (n = 31), and failed collection attempt (n = 19). For 30 cadavers, specimens were of insufficient quality for HIV testing. Of the 610 (75.6%) with an HIV test result available from postmortem blood specimens, 357 (58.5%) had a recorded cause of death. The overall unadjusted HIV-positivity rate among the 610 cadavers was 19.5% (95% confidence interval [CI] = 16.5%–22.9%, or 119 cadavers), and HIV positivity differed significantly by sex (14.6% among men versus 29.5% among women; p<0.001). Cadavers from KNH had significantly higher HIV positivity rates (23.2%; 95% CI = 19.3%–27.7%) than cadavers from Nairobi City mortuary (12.6%; 95% CI = 8.8%–17.8%; p<0.001). After standardizing for the age and sex distribution of expected deaths in Nairobi (*30*), the estimated HIV-positivity rate among adult deaths in Nairobi was 20.9% (95% CI = 17.7%–24.6%). The standardized mortality ratio of deaths among HIV-infected versus HIV-uninfected adults was 4.35% (95% CI = 3.67%–5.15%). The standardized ratio is a relative measure that divides the mortality rate for the infected population by the mortality rate for the uninfected population after standardizing to the age and sex distribution of the infected population. Among hospitalized persons with HIV infection who died at KNH and had available medical files (n = 90), 24.4% (95% CI = 16.5%–34.6%) had received a diagnosis before admission, 22.2% (95% CI = 14.7%–32.2%) had received a diagnosis during hospitalization, and a total of 53.3% (95% CI = 42.8%–63.5%) had not received a diagnosis before death.

## Evaluation of the Surveillance System

To assess the performance attributes and identify the strengths and weaknesses of the HIV-related mortuary surveillance system pilot, CDC conducted an evaluation in November 2015, using the CDC guidelines for evaluating public health surveillance systems (*31*). This report presents findings of the evaluation and includes examination of the following attributes of the surveillance system pilot: simplicity, flexibility, data quality (completeness and validity), acceptability, sensitivity, predictive value positive (PVP), representativeness, timeliness, and stability. The evaluation steps included review of the surveillance system protocol, surveillance forms, and SOPs; in-depth interviews with 20 key informants (surveillance system and laboratory staff and representatives from MOH and funding and implementing partners); and review of the surveillance database. Ethics clearance to conduct the evaluation was received in the form of nonresearch determination from CDC and local institutional review board approval.

## Evaluation Findings

### Simplicity

Simplicity of a public health surveillance system refers to both structure and ease of operation (*31*). The pilot included a straightforward system design and sampling approach (including consecutive sampling of cadavers), preparation of written instructions and SOPs* for specimen collection and data collection, and staff trainings on specimen and data collection. Training included a 1-day classroom instruction session for staff involved in specimen collection and was led by the study coordinator, investigators (epidemiologists and a pathologist), and a biosafety expert. Classroom training was followed by practical exercises in specimen collection and management at the KNH mortuary, which were overseen by a pathologist. Despite these measures, initial implementation of the pilot was complicated by lack of adherence to the inclusion criteria by the mortuary staff, the need to immediately transport mortuary specimens collected after hours and on weekends to the laboratory, manual filling of several surveillance forms in addition to routine paperwork, and difficulty in finding patient files for data extraction in the KNH mortuary. MOH leadership and surveillance system investigators addressed these difficulties by meeting with mortuary and laboratory staff and clarifying specimen collection and SOPs, providing supplemental remuneration for surveillance-related overtime, and suggesting improvements for communication between the mortuary and laboratory staff.

### Flexibility

Flexibility of a public health system refers to its ability to accommodate changes in information needs or operating conditions with little additional time, personnel, or allocated funds (*31*). After implementation, multiple changes were made that reflected the flexibility of the surveillance system to respond to change quickly. Changes to the specimen collection and delivery methods included the size of the needle used to collect cardiac blood (i.e., changed to a longer needle to reach the heart cavity) and the timing of specimen collection after cadaver admission. Additional changes were made to data collection, extraction steps, and variables extracted from patient files. Specimen collection was changed to occur after cadaver registration and before cold storage to increase the likelihood of successful collection. Hemolyzed blood specimens were discarded initially by staff but were included later because the quality of cadaver blood did not affect the quality of HIV test results. All these changes were made easily and did not lead to any major system delays or use of additional resources.

### Data Quality

Data quality reflects completeness and validity of the data (*31*). Steps to ensure data quality collected from the system included consistency checks in the database so that added observations were within the expected ranges, investigation of missing or inconsistent data, and internal quality assurance procedures for HIV testing. Completeness of variables was high with exception of cause of death, which was only available for approximately half of the cadavers, and viral load, which was available for 68% of those that tested positive (Table 1). Cause of death was difficult to obtain from cadavers of persons who did not die in a hospital and if an autopsy had not been performed.

**TABLE 1:** Completeness of select data elements within the HIV-related mortuary surveillance system pilot in Nairobi, Kenya — two sites, January 29–March 3, 2015

Variable	No. with available data	Total no.	% with complete data
Date of admission to mortuary	807	**807**	100.0
Estimated age* or date of birth	807	**807**	100.0
Sex	807	**807**	100.0
Cause of death^†^	357	**807**	44.2
Specimen available	640	**807**	79.3
HIV test result^§^	610	**640**	95.3
Viral load^¶^	81	**119**	68.1

**Abbreviation:** HIV = human immunodeficiency virus.

* Age was estimated by mortuary attendants and later updated from the death notification or medical records, if available.

^†^ Cause of death was abstracted from the death notification form completed by the attending physician for hospital-based deaths at Kenyatta National Hospital (KNH), from the autopsy report completed by the pathologist, or from the archived medical records for hospital-based deaths at KNH.

^§^ Denominator is all deaths with viable specimen collected.

^¶^ Denominator is all deaths with HIV-positive test result.

### Acceptability

Acceptability reflects the willingness of persons and organizations to participate in the surveillance system (*31*). This evaluation assessed the acceptability of the surveillance system only among the mortuary and laboratory staff and did not assess the acceptability of the system by the community. Mortuary staff acceptance was initially poor because collecting specimens from cadavers increased staff workload. MOH leadership helped staff understand the importance of the system and the benefits to public health. MOH staff also helped build relationships between mortuary personnel and various implementing partners. In addition, mortuary staff were compensated for surveillance-related overtime. The compensation was approximately $3.50 per day during the specimen collection period. Initially, laboratory staff also had concerns about the timing of the specimen arrival. This difficulty was overcome by ensuring that mortuary staff kept laboratory staff updated on anticipated specimen arrival times. The evaluation findings suggest that staff were more willing to participate as long as they were compensated for additional work responsibilities.

### Sensitivity and Predictive Value Positive

In the evaluation of a traditional disease surveillance system, sensitivity refers to first, the proportion of cases of a disease in the monitored population detected by the surveillance system and second, the ability to monitor changes over time; PVP is the proportion of reported cases that actually have the health-related event (*31*). The pilot had two main objectives: 1) to determine HIV positivity among cadavers at two mortuary sites in Nairobi, Kenya, and 2) to determine annual cause-specific and HIV-specific mortality rates among the cadavers. Estimating sensitivity and PVP for the system was not possible in the traditional surveillance sense because no existing system identifies the actual number of HIV cases or deaths caused by HIV infection among the general Kenyan or Nairobi population. However, model-based estimates from the Spectrum modeling package, which is supported by the Joint United Nations Programme on HIV/AIDS (UNAIDS), are available for HIV positivity and proportion of deaths caused by HIV in Nairobi. These estimates can serve as a proxy for actual number of cases among the population (*32*).

The surveillance system found higher overall estimated HIV positivity than the Spectrum model (*28*). Female cadavers had higher positivity rates than male cadavers, whereas the Spectrum model predicts higher postmortem HIV positivity among men than women (because of the lower estimated coverage of ART among men than women) (Table 2). The surveillance system also indicated a greater overall proportion of deaths caused by HIV (*29*).

**TABLE 2:** Comparison of HIV-related mortuary surveillance system pilot in Nairobi, Kenya, and UNAIDS adjusted estimates using Spectrum modeling software

Characteristic	Adjusted surveillance system pilot estimates (%)	UNAIDS Spectrum estimates (%)
HIV positivity among cadavers	20.9	11.4
Men	14.8	13.8
Women	30.2	8.3
Deaths caused by HIV	12.6	8.4
Men	6.6	10.9
Women	25.4	5.3

**Sources:** Young PW, Kim AA, Wamicwe J, et al. HIV-associated mortality in the era of antiretroviral therapy scale-up—Nairobi, Kenya, 2015. PLoS One 2017;12:e0181837; Nyagah L, Young PW, Kim A, et al. HIV-related deaths in Nairobi, Kenya: results from a HIV mortuary surveillance study, 2015. J Acquir Immune Defic Syndr. In press.

**Abbreviations:** HIV = human immunodeficiency virus; UNAIDS = Joint United Nations Programme on HIV/AIDS.

### Representativeness

Representativeness compares the characteristics of reported events to all such actual events (*31*). The two largest mortuaries in Nairobi were included in the surveillance system. Of the 12,796 deaths among persons aged ≥15 years registered in Nairobi County in 2014, KNH and Nairobi City mortuaries together registered 54.2% deaths (n = 6,930) (*33*). However, the surveillance system might not be representative of all deaths occurring in the city for several reasons. Some deaths recorded in the system might not have taken place in Nairobi. Conversely, although a death might have occurred in Nairobi, the cadaver could have been taken to a facility outside the city that was closer to the location of death. One of the mortuaries was a hospital mortuary, where the HIV positivity rates are expected to be higher than for the general population because persons who are HIV positive are expected to visit health care facilities more frequently due to illness resulting from HIV infection. This was reflected by the higher HIV-positivity rate reported from the KNH mortuary than from the Nairobi City mortuary. Because KNH is a national referral facility, KNH likely included deaths of non-Nairobi residents transferred from facilities outside Nairobi. Although residency status was recorded, place of residence is not always certain because in Nairobi, persons might misreport their place of birth (e.g., an ancestral home) instead of place of residence. This could be a concern when reporting the mortality rate, which uses the resident population as a denominator and therefore should only include deaths among the resident population as a numerator. Finally, the surveillance system only collected data for 1 month and could not account for potential seasonality in mortality rates or associations between season and cause of death or HIV positivity among persons who died.

### Timeliness

Timeliness refers to the speed between steps of the surveillance system (*31*). Data collection began approximately 3.5 months after approval of the protocol. Once the surveillance system pilot was implemented, specimen collection, laboratory testing, data extraction, and data entry were timely. Data were received by the surveillance team as anticipated without major delays. Timeliness of specimen collection was a concern because delays can affect specimen quality. The target of specimen collection was set within 48 hours of death; however, this evaluation could not confirm whether specimens were collected within 48 hours of death because time of death was rarely documented. The median time between specimen collection and sample receipt at the laboratory was approximately 1 hour (65.5 minutes [interquartile range: 41.5–100.5 minutes]). After data collection was complete, it took about 6 months to enter and clean the data set to prepare it for analysis. This was mainly because of human resource constraints, which delayed data analysis and dissemination of findings. The data collection was completed in March 2015, and the final report was published in July 2016 (*33*).

### Stability

Stability refers to the reliability and availability of the system over time (*31*). Implementing the surveillance system at two mortuaries over a 33-day period cost approximately $50,000. This cost included all study-specific consumables, laboratory testing, salaries for surveillance system pilot staff, and logistics but excluded salaries for mortuary and laboratory staff. These costs could be lower for a replication or continuation of the pilot. Costs can further be lowered if testing can be done on oral saliva specimens, which has not been validated yet but is planned to be implemented in the next surveillance system replication in Kenya. Additional compensation was provided to the current mortuary staff, but no additional staff was hired for the pilot at the mortuaries. The cost for a national system has not been estimated; however, expansion to five urban areas (each with two mortuaries) might cost approximately $250,000, which could be spread over several calendar years to allow for staggered implementation. The surveillance pilot system was relatively inexpensive compared with other HIV surveillance systems in Kenya. For example, the 2012 Kenya AIDS Indicator Survey (*34*), which was a national survey rather than a local system, cost approximately $7.5 million. However, the expansion or replication of the surveillance system will depend at least in part on funding from sources external to MOH because mortality surveillance is not incorporated into the national AIDS strategic framework or the national health policy (*24*).

## Discussion

This report describes the evaluation and certain attributes of an HIV mortuary surveillance system pilot in Nairobi, Kenya. The evaluation indicated that although the system was complex to implement, it was flexible and accommodated multiple changes. In addition, in spite of compensation for the staff involved, the system was relatively inexpensive to implement compared with other surveillance systems. However, the replication or expansion of the system depends on external funding.

The main strength of the evaluation is the breadth of the assessment. As part of the evaluation, in-depth, face-to-face interviews were conducted with surveillance staff (including mortuary and laboratory staff) at all levels and with various stakeholders involved in the funding or implementation of the pilot. This evaluation is subject to at least two limitations. First, sensitivity and PVP of the system were unable to be measured because of lack of data on actual number of cases among the population. Second, cause of death for the majority of cadavers included in the pilot could not be independently ascertained (i.e., person who died had HIV infection versus death caused by HIV infection).

 The system provides unique information on HIV mortality that is not available from other sources in many countries and can help determine epidemic control, which is defined as the point at which new HIV infections decline below the total number of deaths among HIV-infected persons (*35*). However, the UNAIDS goal to end AIDS by 2030 through the 95–95–95 targets (i.e., 95% of persons living with HIV know their HIV status, 95% of persons who know their status receive treatment, and 95% of persons receiving treatment have suppressed viral loads) does not account for mortality among persons infected with HIV (*36*). The lack of global HIV mortality data hinders the measurement of epidemic control. The findings from this pilot suggest that a mortuary surveillance system can provide these data. Because most sub-Saharan African countries lack HIV-related mortality data, an ongoing mortuary surveillance system could be considered.

Data from mortuary surveillance also can be used to calibrate the UNAIDS Spectrum models. In countries that have strong vital registration and disease reporting systems, HIV/AIDS-related mortality data are used to estimate modeled trends in Spectrum (*37*). However, because most sub-Saharan African countries do not have strong systems, Spectrum models rely on other (nonmortality) data to produce mortality estimates, which are subject to the robustness of the data used. Thus, inclusion of mortuary surveillance data in Spectrum models would improve national HIV mortality estimates.

In addition to mortuary surveillance, implementing simple automated procedures for tabulating cause of death when deaths are registered could help Kenya improve the robustness of mortality indicators. To obtain accurate cause of death tabulations that are comparable across geography and time, all deaths should be attended by a health practitioner and *ICD-10* codes should be used for coding all deaths attended (*38*). This would require *ICD-10* coding training for all relevant staff.

Kenya, with support of U.S. President’s Emergency Plan for AIDS Relief (PEPFAR) funding, plans to replicate this mortuary surveillance system beginning in certain regions. For example, replication of this pilot in Western Kenya will provide important data about HIV-associated mortality in a high-prevalence region. As part of the replication, HIV tests also will be conducted on oral fluid specimens and assessed against the blood specimen. The use of oral fluid for HIV testing will allow for noninvasive testing, the addition of children in future studies (because collecting cardiac blood from child cadavers is difficult), and, potentially, the ability to test preserved bodies.

## Conclusion

The HIV-related mortuary surveillance system provided data on HIV positivity among cadavers and HIV-associated mortality, which are not readily available from other sources. The system can illuminate HIV-related mortality by providing ongoing continuous data on HIV positivity among cadavers and annual HIV-proportional mortality rates. Availability of mortality data can help describe a country’s progress toward achieving epidemic control. A mortuary surveillance system paired with improved cause of death reporting can provide important insights into HIV epidemic trends.

## Footnotes

* SOPs can be requested from Peter Young, CDC Kenya (fqm1@cdc.gov).
